# Opportunities for artificial intelligence and synthetic biology in designing living drug delivery systems^☆^

**DOI:** 10.1016/j.addr.2026.115883

**Published:** 2026-04-27

**Authors:** Jason H. Yang, Caleb J. Bashor

**Affiliations:** a Center for Emerging and Re-emerging Pathogens, Rutgers New Jersey Medical School, Newark, NJ 07103, USA; b Department of Microbiology, Biochemistry, and Molecular Genetics, Rutgers New Jersey Medical School, Newark, NJ 07103, USA; c Department of Bioengineering, Rice University, Houston, TX 77005, USA; d Department of Biosciences, Rice University, Houston, TX 77005, USA; e Rice Synthetic Biology Institute, Rice University, Houston, TX 77005, USA; f Ken Kennedy Institute, Rice University, Houston, TX, 77005, USA

**Keywords:** Living drug delivery, Artificial intelligence, Synthetic biology, Structure-to-function, Composition-to-function, Massively parallel reporter assays

## Abstract

Advances in artificial intelligence (AI) and synthetic biology are transforming biological research and biotechnology. These fields are for the first time enabling the design and development of human and bacterial cells that can serve as “living” drug delivery vehicles that perform sustained release of therapeutic cargo with spatiotemporal control. In recent years, human and bacterial cells have been engineered to deliver peptides, proteins, and biologics for treating a wide range of human diseases using synthetic biology approaches. To engineer effective living drug delivery systems, detailed knowledge is required about how to design receptors that can specifically sense the tissues targeted for drug delivery, signaling networks that can process signals from these receptors, and gene circuits that can control therapeutic cargo production and release. However, elucidating such receptors, signal processing and gene regulatory elements, and gene circuit compositions by traditional design- build-test-learn approaches is difficult and low throughput. Here we review how advances in AI and synthetic biology are meeting these challenges. We describe examples of how human cells and bacteria are engineered to become living drug delivery vehicles. We discuss how AI and synthetic biology approaches are being applied to discover the sequence-to-function design principles for engineering synthetic receptors, signaling proteins, and gene regulatory elements and the composition-to-function design principles for engineering synthetic gene circuits. We share an outlook on opportunities for AI and synthetic biology to synergize for creating next-generation living drug delivery systems.

## Graphical Abstract

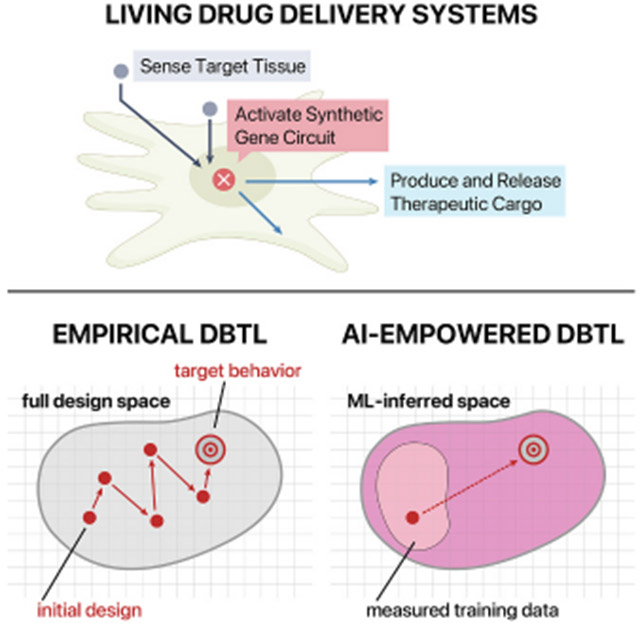

## 1. Introduction

Over the past 25 years, parallel advances in synthetic biology and artificial intelligence (AI) have transformed both basic biological research and biotechnology [1]. While drug discovery, development, and delivery are historically rooted in the traditions of chemistry, biology, and material science, advances in genetic engineering and machine learning (ML) are accelerating the discovery and development of innovative therapies for treating complex diseases such as cancer and cardiovascular disease. Much of this attention has focused on using synthetic biology and artificial intelligence approaches to discover and develop drugs such as small molecules, peptide or protein biologics, and nucleic acid technologies (e.g., mRNA vaccines, CRISPR-based genome editors). However, the utility of such drugs to treat human diseases is limited by the challenge of delivering them with specificity and spatiotemporal control while limiting toxicity. Recent advances in protein, peptide, and lipid engineering are increasingly demonstrating that contributions from the domains of biology and genetics can complement the contributions by chemistry and material science. In parallel, nextgeneration drug delivery systems that utilize genetically tractable organisms such as human cells and bacteria as delivery vehicles are coming online [2-8]. Thus, there is a significant opportunity for synthetic biology and AI to transform drug delivery. Here we review recent advances in applying synthetic biology and AI approaches to engineer next-generation drug delivery systems. We highlight opportunities for continued development in the near and long-term horizon.

## 2. Synthetic biology

**Fig. 1.**
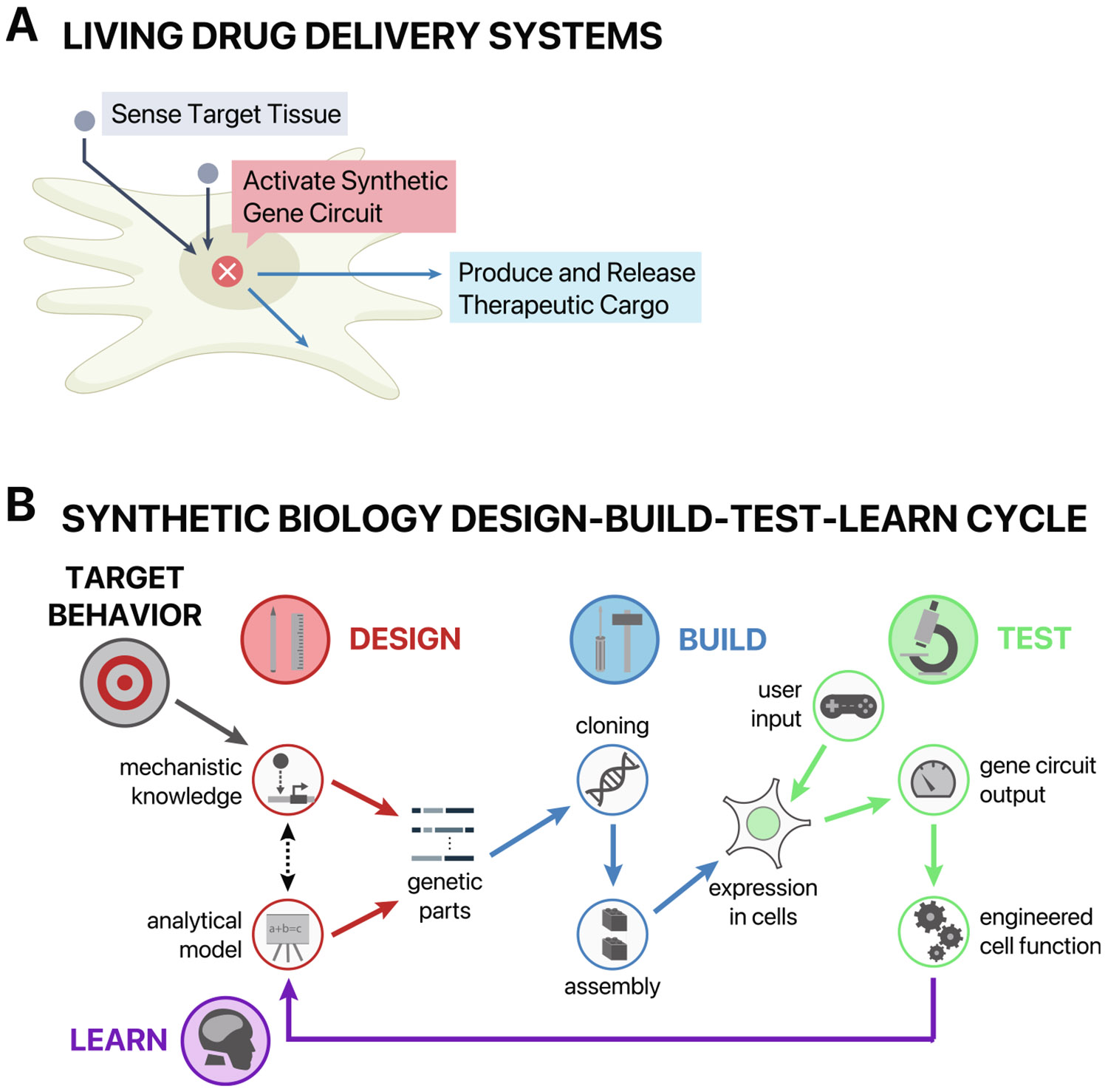
Living drug delivery systems can be engineered using synthetic biology approaches. (A) Human and bacterial cells are now being engineered to function as living drug delivery vehicles. These cells are designed to perform sustained release under spatiotemporal control by sensing tissue targets and activating synthetic gene circuits that induce programmable production and release. (B) The design-build-test-learn (DBTL) cycle is an empirical framework used in synthetic biology to optimize the design and engineering of synthetic gene circuits.

Synthetic biology is an engineering discipline that uses DNA-based programming to create biological systems that fulfill user-specified design objectives [9-12]. Historically, synthetic biology emerged from the fields of genetics, which studies how genes and associated regulatory elements encode cellular behavior, and enginering, in which complex systems are manipulated and studied using quantitative mathematical models [9,13-15]. The combination of these research domains gave rise to the rational engineering of gene circuits, in which gene regulatory elements (e.g., promoters) and expression machinery (e.g., transcription factors [TFs]) are engineered to program expression dynamics in response to external cues. This, in turn, enabled engineered cells with behaviors controlled by embedded “gene circuits” and, more recently, cell-based therapeutics in which cells are programmed to execute therapeutic functions in response to disease-associated signals [6,8,12,16-23] (Fig. 1A).

As with the development of drug delivery systems, synthetic biology has traditionally followed a design-build-test-learn (DBTL) paradigm (Fig. 1B). In this framework, gene regulatory elements (“parts”), gene circuits, and cells are rationally designed based on defined objectives, prior knowledge, and first principles-based mathematical modeling. Parts are synthesized and assembled using molecular biology approaches and introduced into appropriate cellular chassis (e.g., T cells). The performance of the engineered system is quantitatively assessed, and the resulting data are used to train and refine mathematical and conceptual models to learn principles that inform the next DBTL round.

In practice, the DBTL process is slow, labor intensive, and low throughput due to limitations in the ability to synthesize and evaluate engineered systems at scale. However, over the last 5–10 years, advances in oligonucleotide synthesis, next-generation sequencing (NGS), and ML have started to break this bottleneck [24-38], accelerating the discovery of sequence-to-function and composition-to-function design principles for engineering genetic parts, gene circuits, and cells. AI-driven sequence-to-function studies have, for example, enabled discovery of synthetic promoters [39-41] and enhancers [42-44]. Examples of composition-to-function discovery include AI-driven discovery of gene circuit part [45] and chromatin regulator [46] assemblies and chimeric antigen receptor (CAR) signaling domains [47,48] that yield predictable circuit behaviors.

## 3. Artificial intelligence

AI is a computer science discipline that applies trained ML models to perform complex tasks with human-like intelligence. ML models utilize large quantities of training data to “learn” the patterns and quantitative relationships between input “features” and output “labels”. Once trained, ML models can predict unobserved labels based on user-supplied features. The algorithms that underlie ML are premised on statistics, information theory, and data transformation, and can operate on a wide range of input or output data types, including multidimensional, discrete, continuous, categorical, graph, and time-series data [49-52]. The recent boom in advanced large language models [53,54] was a watershed moment that is transforming human society.

In recent years, AI approaches have been advancing biomedical research at a dizzying pace [55,56]. For the first time, researchers are leveraging large language models to form “foundation models” that integrate sequence information at the DNA, RNA, and/or protein levels to predict biochemical, cellular, and/or molecular function [57-62]. Conceptually, these foundation models “learn” fundamental rules that relate biological sequence information to observable function, even when specific molecular mechanisms have not yet been discovered. These models can be leveraged for the de novo design of biological parts such as proteins with specified enzymatic activities, DNA regulatory elements, and RNA-based regulatory devices such as synthetic riboswitches. Thus, biological foundation models can be used to design both synthetic gene circuits and effector proteins. Such models are already being employed by synthetic biologists for de novo protein or genome engineering [58,59]. Thus, they hold significant potential for designing living drug delivery systems with user-defined capabilities.

AI and ML are increasingly utilized for drug delivery research and development [63]. The application of AI to chemistry- and materials- based delivery systems is extensively reviewed elsewhere in this issue of *Advanced Drug Delivery Reviews*. Here we specifically focus on how AI and synthetic biology are advancing the development of human and bacterial cell-based drug delivery systems, how AI and synthetic biology are transforming cell engineering at large, and how AI and synthetic biology can be applied to further advance the engineering of living drug delivery systems.

## 4. “Living” cell-based drug delivery systems

The ideal drug delivery system achieves specific and localized delivery of therapeutic cargo to diseased tissues over timescales relevant for treating disease [64]. Traditional drug delivery systems achieve this goal by taking advantage of advances in chemistry and material science to create formulations and materials that possess desired targeting and release properties. However, targeted delivery remains hindered by intravascular, endothelial, extracellular, and cellular barriers [5,7]— obstacles that evolution has already enabled certain cells types, such as immune cells, to overcome. Moreover, sustained delivery of therapeutic payloads is often constrained by limited capacity and controlled-release properties. Drug delivery vehicles that continuously synthesize therapeutic molecules—or take advantage of a patien’s cells to do so—can overcome these bariers to bioavailability. These challenges have motivated the development of “living” cell-based therapies and drug delivery systems [2-8].

A key advantage of working with cell-based delivery systems is that cells have evolved the capacity to sense complex environmental signals and to respond with high specificity and spatiotemporal precision [6,16,65]. These capabilities are mediated by surface protein receptors that are activated by extracellular cues and processed by intracellular signaling, metabolic, and gene regulatory networks to elicit defined behaviors. Because these processes are genetically encoded, they can be reprogrammed using synthetic biology tools to express factors that redirect cellular function via highly specific, high-affinity interactions with markers located in diseased tissue. In addition, many therapeutic cell types are motile and can home to sites of inflammation, tissue injury, or tumor growth in response to disease-associated cues. Cells can function as living factories, continuously synthesizing therapeutic biomolecules such as proteins, peptides, and nucleic acids, thus maintaining local drug levels without the need for repeated dosing. Cellular drug delivery systems can be engineered to be minimally immunogenic, reducing immune clearance and adverse inflammatory responses. These traits collectively make cells excellent candidates for delivering a wide range of therapeutic payloads.

### 4.1. Human cells as drug delivery vehicles

Many human cell types possess traits that are attractive for drug delivery. They perform a wide range of cell type-specific functions and their roles in human health and disease are well understood at a high level. Moreover, they also possess cell type-specific properties that can be useful in diverse applications. For example, autologous or type-matched erythrocytes are minimally toxic and can be loaded with large quantities of therapeutic cargo without complex genetic engineering, and can continuously release their cargo over long timescales [66,67]. Naïve and engineered erythrocytes have been used to deliver a wide range of drugs including small molecules such as dexamethasone [68] and enalaprilat [69], peptide nucleic acids [70], antigens [71,72], and therapeutic enzymes such as L-asparaginase [73] and reteplase [74]. However, because erythrocytes lack a nucleus, genetic engineering tools for them are poorly developed. Thus, synthetic biology activities in these cells have been limited (Table 1).

Immune effector cells (e.g., T cells, NK cells, and macrophages) have recently gained pervasive interest as drug delivery vehicles. Their natural abilities to traffic to diseased tissues, perform complex signal processing, move across the blood brain barrier, and perform therapeutic functions have made them attractive substrates for cell-based therapy [16,21,22,65,75-78]. In particular, cytotoxic T and NK cells have been preferentially functionalized with synthetic chimeric antigen receptors (CARs) to home to tumors and kill cancer cells [79]. The premise for this technology is that CARs can be synthetically engineered to specifically recognize user-defined targets and that gene circuits can be engineered to elicit user-specified cell functions upon CAR activation. CAR-T cell therapies have now been extended beyond cancer to treat a wide range of complex diseases including cardiac fibrosis [80] and autoimmune disorders [81].

As CAR technologies have matured, there is growing interest in functionalizing their stimulatory release properties to deliver immunomodulatory cytokines [82]. Macrophages are becoming exciting substrates for CAR-based therapeutics [83-88], owing to their high plasticity, large repertoire of receptors that sense diseased-associated markers, and long history as vehicles for delivering antigens, immunomodulatory cytokines, nanoparticles, small molecule drugs [76,77,89-92]. CAR-macrophages have been developed to treat myocardial ischemia-reperfusion injury [93,94], and kidney ischemiareperfusion injury [86], and cancer [84,95,96]. Co-culture experiments have revealed that engagement of CAR-macrophages with tumor cells can induce release of proinflammatory cytokines [84,95]. The therapeutic benefit of CAR-macrophages against kidney ischemic- reperfusion injury involved induction into an anti-inflammatory state associated with the release of anti-inflammatory cytokines [86].

These examples illustrate how human cells with distinct intrinsic properties and activatable behaviors can be engineered to deliver natural and synthetic cargo. Human cells natively possess machinery for environmental sensing and signal processing which may be genetically engineered for controlled release in response to disease-associated markers, making them ideal candidates for closing the drug-delivery specificity gap. Their larger volumes compared with bacteria and most non-living drug delivery vehicles, enables them to deliver larger quantities of therapeutic cargo (either preloaded or continuously translated) over their residence time in diseased tissues, thereby mitigating the drug bioavailability gap. However, because engineered human cells retain most of their cell type-specific properties, problems can arise when non-programmed behaviors are activated in vivo. Thus, a central challenge for human cell drug delivery is to ensure that mechanisms underlying controlled release of therapeutic cargo are insulated from, or made compatible with, the endogenous signaling and gene regulatory networks of the selected human cell chassis.

### 4.2. Bacteria as drug delivery vehicles

Bacteria are metabolically and physiologically versatile and were the first organisms used in synthetic biology research [9,13-15]. In *Escherichia coli* and many other bacteria, synthetic biology toolkits exist that enable rapid design and assembly of synthetic gene circuits [97-104]. Moreover, most genetically tractable bacteria are well-studied, with gene regulatory [105-110] and metabolic [111-114] networks that are well characterized. Together, these features support the rational engineering of bacteria with complex phenotypes [115], and have made them alternative candidates for controllable release of biologics such as peptides, proteins, and nucleic acids (Table 2).

Bacteria also play important roles in regulating health and disease as part of the human microbiome [144-146]. Commensal bacteria support human health by secreting natural products and metabolic byproducts that act on human cells. These interactions have motivated the widespread use of probiotics, prebiotics, postbiotics, and synbiotics as health adjuvants [147-149]. Because commensal bacteria are biologically tolerable, engineered commensals are frequently used as substrates for cell-based therapies [3,6,8,20].

Most studies functionalizing bacteria for drug delivery have focused on gastrointestinal commensals due to their safety profiles. For example, *E. coli* has been engineered to deliver phenylalanine-degrading enzymes [118], antimicrobial peptides [119], GLP-1 receptor agonists [120], and nanobodies that neutralize CD47 [121] and TNFα [122] to treat a wide range of diseases from Mendelian disease, to gastrointestinal infection, to complex diseases such as diabetes and cancer, respectively. These examples demonstrate how bacterial physiology can be uniquely functionalized to imbue living drug delivery vehicles with properies difficult to achieve in engineered human cells or traditional drug-delivery vehicles, such as self-amplifying delivery of therapeutic cargo [119] and theranostic controlled release [121]. Other examples include *Lactococcus lactis*, which has been engineered to deliver IL-10 [123], β-lactamases [124], and TNFα-neutralizing nanobodies [125] to treat inflammatory bowel diseases and VEGF [126], and the combination of FGF-2, IL-4, and CSF-1 [127] to enhance wound healing; and *Bifidobacterium longum*, which has been engineered to deliver oxyntomodulin to reduce obesity [150], manganese superoxide dismutase [116] to suppress colitis, and HER2-inhibitory antibodies [117] to inhibit tumor growth. Many examples exist for several other commensal bacteria [6,8,101,151].

Pathogenic bacteria have also emerged as an important target space for engineering drug delivery vehicles [8]. Many pathogens co-evolved with humans and possess secretion systems that enable effective delivery of virulence factors and other proteins [152-155]. Pathogenic bacteria also elicit immune responses that may be effective for treating diseases such as cancer [20,156]. However, in most cases, pathogenic bacteria must first be attenuated by removing virulence genes before they can be utilized as therapeutics. Examples of pathogenic bacterial engineered as drug chassis include the intracellular pathogen *Salmonella enterica*, which has been engineered to deliver viral antigens as oral vaccines [136] or cytotoxic Cp53 [137], oncolytic viruses [138], and immunomodulatory CCL21 [139], GM-CSF and IL-7 [140], IL-18 [141], and IL-2 [142] as cancer therapies. In addition, the opportunistic lung pathogen *Pseudomonas aeruginosa* has been engineered to deliver the transdifferentiating transcription factor MyoD [132] and the tumor-associated antigens ovalbumin [133] and TRP-2 [134]. Other examples include *Mycobacterium bovis BCG*, *Listeria monocytogenes*, and *Yersinia enterocolitica* [6,8].

Components of non-human-associated bacteria have also been explored into as drug delivery machinery. Bacteria living in diverse environmental contexts have evolved specialized mechanisms for adapting to their environments. Such mechanisms include bacterial secretion systems, which are contractile syringe-like complexes that resemble bacteriophage tails and that are found in many environmental bacteria [157]. These systems enable intracellular delivery of proteins and nucleic acids into neighboring cells. Recently, *E. coli* was engineered to heterologously express a contractile injection system from the ento-mopathogen *Photorhabdus asymbiotica* [158,159]. This enabled delivery of a diverse range of payload substrates into mouse and human cells, including toxin proteins and several genome editing molecules like toxin proteins, ribonucleoproteins, and single-stranded DNA.

*Mycoplasma pneumoniae* recently arose as an attractive candidate for drug delivery [160]. It possesses a reduced genome of 816 kb and lacks genes for several biosynthetic pathways [161]. *M. pneumoniae* possesses a doubling time of ~20 h [114] and also lacks a cell wall which enables immune evasion [162] and rapid release of translated proteins [160]. Moreover, attenuated *M. pneumoniae* can survive at least 4 days in a mammalian host without pathogenicity before clearance by the liver or immune system [128]. *M. pneumoniae* has been engineered to express single-chain IL-10 [128] and biofilm dispersing enzymes [129-131]. These were demonstrated to suppress inflammation and lung infection in mouse models of disease.

A unique advantage of bacterial drug delivery vehicles is that engineered bacteria can be programmed to lyse following exogenous or endogenous cues, a property that enables controlled release of therapeutic cargo and controlled maintenance of bacterial population sizes. For example, gene circuits have been engineered into *E. coli* to induce cell-density dependent auto-regulatory lysis [121,143,163]. This strategy was employed to engineer *E. coli* cells that delivered CD47-neutralizing nanobodies to syngeneic tumors in mice [121]. *P. aeruginosa* cells have also been engineered to deliver antitumor Hemolysin E to A549 tumors in mice upon optogenetic lysis [135].

Thus, like human cells, bacteria can be engineered to controllably deliver biosynthetic therapeutics. However, it is challenging to engineer mechanisms that allow bacterial to sense diseased tissue since their cell membranes and signaling architectures significantly differ from that of human cells. Consequently, receptors engineered for human cells do not function reliably in bacterial hosts. Moreover, bacterial drug chassis risk eliciting unwanted immune responses. Despite these limitations, the extensive genetic toolkits available for bacteria create opportunities to engineer bacterial delivery vehicles to possess greater complexity and diversity in processing external signals to control therapeutic release.

## 5. Artificial intelligence and synthetic biology in living drug delivery system design

**Fig. 2.**
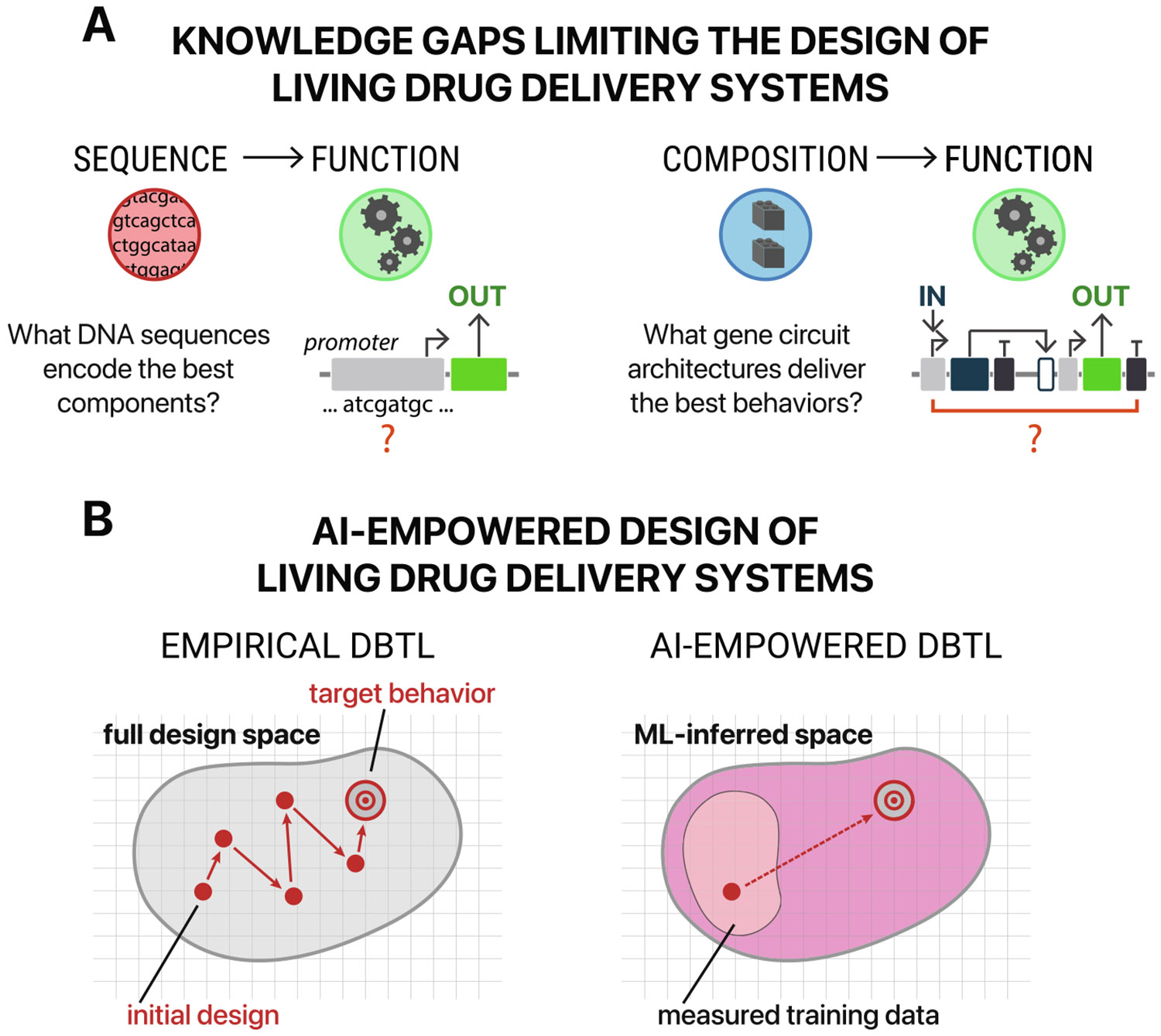
Artificial intelligence is accelerating the design of living drug delivery systems. (A) Living drug delivery system design is limited by knowledge gaps in the sequence-to-function relationships needed to engineer synthetic targeting receptors and gene regulatory elements and composition-to-function relationships needed to select and arrange synthetic gene circuit components. (B) Traditional DBTL approaches are slow, incremental, and low throughput. Advances in high throughput oligonucleotide synthesis, next-generation sequencing, and machine learning are enabling rapid AI-empowered discovery of design principles for engineering synthetic components, gene circuits, and cells with predictable and programmable behaviors.

The application of AI and ML to advance drug delivery system design is still at an early stage [164]. Advances in synthetic biology are now enabling creation of next-generation living drug delivery systems with improved sensing, processing, production, and controlled release properties [16]. Realizing these capabilities requires more information on how machineries supporting these functions can be genetically engineered. These knowledge gaps can be subdivided into gaps in understanding the genetic sequence-to-function relationships for sensory, signal processing, and gene regulatory elements and gaps in understanding the composition-to-function relationships that govern how these components can be assembled (Fig. 2A). Closing these gaps is currently rate-limited by iterative DBTL cycles. However, the rise of AI, next-generation sequencing (NGS), and high throughput oligonucleotide synthesis technologies are transforming the pace at which the datasets required for AI model training can be generated and at which the analyses for learning design principles can be performed [24,26-37] (Fig. 2B).

### 5.1. Sequence-to-function relationships for environmental sensing

A useful framework in synthetic biology for understanding and engineering cells is “sense-process-respond”. Under this framework, cells receive information from extracellular signaling receptors (sense) and interpret this information using intracellular signaling and gene regulatory networks (process) to selectively activate different cellular functions (respond). Current strategies for engineering cells that sense environmental cues utilize either naturally occurring receptors or fragments of antibody fragments. However, these strategies significantly limit the environmental sensing capacities of drug delivery vehicles to well-studied targets. The ideal living drug chassis would recognize user-defined disease-associated markers and trigger a signal processing cascade, even if those target markers are poorly characterized [165-167]. These necessitate the de novo design of binder proteins that can be engineered into synthetic signaling receptors [168].

De novo synthetic receptor engineering requires the ability to design protein domains that bind to ligands with high affinity and the ability to couple binding to downstream cellular functions. AI has already transformed protein engineering by enabling high-fidelity prediction of 3D structure of proteins and protein complexes directly from amino acid sequences [61,169-182]. For example, one family of AI-driven structure prediction tools is AlphaFold [61,172,173]. The original AlphaFold system used a top-down deep learning model trained on protein sequence and structure data from the Protein Data Bank [183,184] to predict inter-residue distances based on homology to proteins with known structure [172]. Subsequent versions improved upon this framework by jointly modeling sequence-residue and residue-residue interactions to predict the structures of protein complexes and proteins bound to nucleic acids and small molecules [61,173].

Other important protein structure and binding prediction platforms include RoseTTAFold (e.g., RFDiffusion) [175-178], FoldX [185-188], and BoltzGen [169,189,190]. RoseTTAFold predicts protein structures by first jointly integrating ID information about protein sequences, 2D information about residue-residue interactions, and 3D atomic coordinate information and then minimizing the overall free energy state using physics-based energy functions [191-193]. FoldX makes predictions on protein folding and stability by modeling force field-constrained Gibbs free energy differences under different protein conformations. BoltzGen utilizes generative deep learning to atomistically model structures based on probabilistic free energy minimization [169]. These methods have been used to predict structures for proteins complexed with proteins, nucleic acids, metals, small molecules, and covalent modifications [169,178,194,195].

These modeling frameworks are now enabling AI-driven de novo design of “binder” proteins that can bind diverse molecular targets with high affinity [196-201]. In one notable study, two deep learning approaches were employed for designing binder proteins given only information on the structure of a target protein [197]. The first method (“constrained hallucination”) applied trRosetta or RoseTTAFold to randomly sample candidate amino acid sequences to predict proteins that can complex with a target. A multi-objective loss function was defined that scores the structural accuracy of a binding motif at a targe’s functional site and how confident a candidate sequence encodes that backbone. Sequences were iteratively improved to minimize the multiobjective loss function. This enabled prediction of several proteins that bound PD-L1 with mid-nanomolar affinity. The second method (“inpainting”) began with binding protein fragments that do not fully bind the target protein and used RoseTTAFold to predict sequences and structure that can “complete” a binder protein containing these fragments. These two approaches were employed to design proteins scaffolding a diverse set of protein targets including the ACE2 epitope that binds SARS-CoV-2, the iron binding site from *E. coli* bacterioferritin, the enzyme active sites of carbonic anhydrase II, and the native proteinbinding domains of PD-L1 and TrkA.

These examples demonstrate that current AI tools can already design binder proteins for a wide range of protein targets. This is a significant innovation as de novo protein design unlocks the potential for engineering biologically active molecules completely orthogonal to the natural world [168]. For living drug delivery vehicles, this would enable highly sensitive and specific environmental sensing, which would be important for targeting newly discovered or poorly characterized disease-associated markers. We envision that these tools will be critically integrated into living drug delivery vehicle design workflows for engineering synthetic targeting receptors, intracellular signaling proteins, and/or gene regulatory proteins.

### 5.2. Sequence-to-function relationships for signal processing

While the ability to rapidly design protein binders is a major leap forward for synthetic environmental sensing, controlled activation of cellular responses to external cues requires programmable signal transduction from receptor to effector. Bacteria primarily employ simple two-component signal transduction systems to transmit responses to extracellular signals [202,203]. In prototypical two-component systems, activation of a transmembrane histidine kinase sensor induces phosphorylation of its cognate response regulator, which in turn regulates gene expression. Despite their relatively simple architecture, two-component systems can perform complex nonlinear signal processing through phosphorelay feedback regulation [204]. In eukaryotes, signal processing is more complex than in bacteria due to the greater diversity of signal transduction mechanisms [205-209]. Signal transduction is most commonly mediated by post-translational modifications of signaling proteins, and the connectivity among these species gives rise to complex signaling behaviors with emergent properties [210,211].

Signal transduction is an exciting frontier for synthetic biology and early progress has been made in developing technologies that use phosphokinase [212,213] or protease [214,215] activity to regulate cell behaviors. However, signal transduction is difficult to engineer because signaling proteins must both sense and respond to intracellular signaling molecules. This creates two concurrent design objectives for de novo protein design. However, AI-driven protein design tools are beginning to meet this challenge.

A recent study leveraged AlphaFold to design tunable Ras activity biosensors that can actuate cellular responses following Ras-GTP activation (RAS LOCKR) [216]. Development of these biosensing actuators leveraged two prior innovations. First, a technology for engineering latching orthogonal cage-key protein (LOCKR) switches was used, in which the active site of a biosensing protein becomes uncaged upon binding to a target “key” protein [217]. LOCKR variants which degrade target proteins upon activation (degronLOCKR) are able to exert tunable signaling feedback control [218]. Second, a technology for engineering actuatable enzyme activity was used, in which an enzyme is split into two proteins that become reconstituted upon LOCKR activation [219]. This prior study created a luciferase protein biosensor comprised of two proteins that each contain a split luciferase. One component is a LOCKR protein (lucCage) that contains half of a split luciferase and the other is a “key” protein that possesses the other half of a split luciferase (lucKey). Target-binding induces a conformational change in lucCage that enables lucKey to complex and the split luciferase to reconstitute, which enabled read out of target protein abundances by luminescence. To determine where target-binding peptides should be engineered, a Rosetta-based method (GraftSwitchMover [217]) was applied to stabilize the luc-Cage closed configuration.

To first construct a LOCKR system that sensed intracellular Ras-GTP, a lucCage protein was engineered to deliver a CFP-YFP FRET-based readout instead of luminescence-based readout. GraftSwitchMover and AlphaFold was used to thermodynamically optimize latch-cage and key-cage interactions with the Ras binding domain from Raf1. Once Ras-GTP sensing was validated, the resulting RAS LOCKR biosensor was further engineered to proximity label neighboring proteins by replacing the FRET fluorescent proteins with a split biotin ligase. This delivered a RAS LOCKR biosensor that actuated proximity labeling in the presence of Ras-GTP—a strategy that can be generalized for other upstream signaling proteins and downstream signaling activities.

Thus, this early example demonstrates how AI can advance the design and development of proteins that can be actuated by signaling molecules to exert enzymatic activities. We envision that AI-driven protein design will enable creation of fully synthetic signaling cascades [212] that are insulated from endogenous signaling networks and that will deliver living drug delivery vehicles with nonlinear activation properties. This would enable living drug delivery vehicles that retain cell type-specific behaviors without non-programmed activation.

### 5.3. Sequence-to-function relationships for gene expression regulation

In many synthetic biology applications, the programmed response of an engineered cell to an exogenous cue is a change in the expression of a target gene. In the case of living drug chassis, this target gene may be a secreted gene product or a biosynthetic pathway. Predictable and programmable control of cellular responses to signaling activation requires well-characterized parts for gene regulatory elements that can be used to engineer synthetic gene circuits. However rational design of such parts is encumbered by the high dimensionality of amino acid and nucleic acid sequence space. Moreover, rational design requires knowledge of the relationships between sequence features and functional behaviors of biological molecules, much of which remains to be uncovered. Efforts like ENCODE reveal how poorly mammalian gene regulation is understood [220-223] and first-principles models for predicting signaling and gene regulatory function from amino acid and nucleic acid sequences alone are lacking.

In recent years, geneticists have developed approaches that integrate high throughput DNA synthesis and assembly, NGS, and ML to develop massively parallel reporter assays (MPRA) to decode the regulatory grammar of mammalian gene expression [30,42,43,224-233]. The conceptual basis behind MPRAs is that large libraries of gene regulatory elements (e.g., promoter or enhancer sequences) can be randomly synthesized with unique barcodes labeling each variant. These libraries are introduced into cells and pooled genetic screens are performed to identify variants with different cellular phenotypes (e.g., expression level of a reporter gene). Top-down ML models are trained using the resulting data are used to predict cellular phenotypes from barcoded sequence features. To date, at least 130 MPRAs have been performed in at least 35 cell types and 4 organisms, revealing significant sequence-feature insights into promoters, enhancers, and cis-regulatory sequences [30,224]. Data from these studies have recently been applied to train ML models to enable predictive design of parts for engineering gene circuits [24]. Numerous examples exist, including synthetic promoters [39-41], enhancers [42-44], programmable RNA switches [234], riboregulators [235], and RNA aptamers [236].

These examples demonstrate how AI activities can be coupled with large-scale genetic screens to elucidate design principles for synthetic gene regulatory elements.

### 5.4. Composition-to-function relationships for signal processing and gene expression

A critical challenge for synthetic biology is the behavioral uncertainty of regulatory circuits assembled from different biological components [237]. This is especially pronounced in eukaryotic cells, where the complexity of gene regulatory and signaling network mechanisms significantly hinders the quantitative predictability of cellular responses to perturbations. Networked interactions among these processes give rise to unexpected nonlinear behaviors that are challenging to model and predict. As a result, selecting the right signaling and gene circuit designs for creating living drug delivery systems with programmable behavior is highly non-intuitive. Moreover, the behavior of genetically engineered modules (e.g., for sensing, processing, production, or release) depends on the overall physiology of the host cell and availability of cellular machineries and resources [238,239]. For example, protein (over)-expression is metabolically costly and the finite availability of ATP, amino acids, and enzymatic substrates constrains the productive yield of biosynthetic processes [240,241]. Thus, metabolic changes in a drug chassis can adversely impact production of therapeutic payloads.

First principles-based modeling tools exist for the predictable design of simple gene circuits, these tools do not scale for circuits with many components [242-245]. Although DNA foundation models that can predict long range gene regulation are now coming online (such as Evo [62], Evo 2 [58], and AlphaGenome [57]), they cannot predict dynamic cell type-specific gene regulation. Moreover, the parts combinations and design permutations for large circuits are typically too expansive to be tested using traditional, low throughput approaches. This has spurred efforts to massively scale the DBTL process by taking advantage of high throughput DNA synthesis, next-generation sequencing, and ML [24,26-37]. Progress has been made in advancing AI-driven circuit design for both synthetic gene and signaling networks.

In one compelling example, a platform was developed for elucidating design principles for engineering inducible gene circuits that maximize fold-change expression (CLASSIC: combining long and short-range sequencing to interrogate genetic complexity) [45]. The authors developed an integrated long- and short-read sequencing workflow to enable construction, validation, and measurement of a libraries of over 100,000 circuit designs. In an initial demonstration, a library of inducible single-input gene circuits composed of synthetic TF-coding and reporter expression units were combinatorially permuted across multiple part categories (e.g., transcriptional activation domains, intrinsically disordered protein domains, TF binding domains, promoters, and terminators). Following a MPRA-like approach, the library was transduced into HEK293T cells and inducible expression was characterized to identify architectures that enabled high fold-change activity. ML learning models were trained to extrapolate findings from this initial screen to the complete design space of over 165,000 circuit compositions. The approach was then extended to determine rules for multi-input gene circuits comprising two synthetic TFs. Using a multi-step process, data were collected to train a model on a “base” design space of ~1.1 million compositions, followed by fine-tuning of the model with “expansion” dataset to map the behaviors of ~3.4 billion circuits. The authors were able to use this approach to identify and experimentally validate circuits with digital-like logic-gated gene expression profiles and infer to infer their underlying design rules.

In a related study, a library of over 1900 chromatin regulator pairs was synthesized and expressed in *Saccharomyces cerevisiae* [46]. Cells were screened for expression of a reporter gene and different ML regression models were trained to predict expression from chromatin regulator co-recruitment. Predictions from this model were experimentally validated by synthesizing out-of-sample gene circuit assemblies. These two examples illustrate how AI approaches can be applied (in a manner like MPRAs) to learn design principles for composing synthetic gene circuits from previously characterized parts to achieve predictable gene expression phenotypes.

In a standout study of signaling systems, CAR activation and co-stimulatory motifs were combinatorially permuted to generate a library of over 2300 receptors [47]. The library was transduced into primary human T cells and screened for cytotoxicity and stemness (associated with T cell persistence). A deep learning model was trained from the resulting data that predicted phenotypes from CAR composition. The model was validated by designing, constructing, and testing a synthetic CAR possessing costimulatory signaling domains predicted by the model to enable enhanced cytotoxic activity. In a follow-up study, the authors probed a design space of over 30,000 synthetic cytokine receptors from an expanded set of intracellular signaling domains and transduced the receptors into primary human T cells [48]. In addition to cytotoxicity, effector memory, and proliferation phenotypes, STAT phosphorylation was characterized in cells expressing the receptors. A deep learning model trained on the resulting data elucidated receptor design principles for selecting synthetic receptor signaling domains that can enable a continuous spectrum of JAK/STAT signaling activities and cytotoxicity, memory, and proliferation phenotypes.

## 6. Hurdles on the path towards clinical translation

Although living drug delivery vehicles possess transformative potential for curing complex diseases, they possess several unique barriers that must all be overcome for clinical translation [16,246,247]. These barriers include challenges to their safety and efficacy profiles that may hinder their path towards regulatory approval and may limit their widespread use and commercial success. AI methods are already beginning to address some of these challenges, but solutions to these hurdles will be nontrivial.

### 6.1. Biological hurdles

Living drug delivery vehicles differ from traditional drug delivery vehicles by their use of biological processes and biochemical interactions to elicit therapeutic benefits. However, this dependence on biology and biochemistry introduces several unique challenges. First, for living cells to persist and function, they must possess metabolic activity and the ability to perform transcription and translation. Thus, the in vivo efficacy of living drug delivery vehicles will depend on the metabolic environment of damaged or diseased tissues. This can be problematic for treating solid tumors or granulomatous diseases such as tuberculosis where the “necrotic core” of a tumor or granuloma is hypoxic and comprised of dead cells [248,249]. Moreover, physical barriers and suppressive signaling cues may hinder trafficking and localization and/or inactivate engineered cells at the site of treatment. Such obstacles are a major source of failure for CAR-T cells in the solid tumor microenvironment [250,251].

Second, living drug delivery vehicles must maintain genetic stability to retain potency. Gene and protein expression is metabolically costly [240,241], which create selective pressures against engineered gene circuits [252]. Moreover, achieving local therapeutic efficacy requires that enough cells are present at the site of treatment over the required treatment duration. Consequently, non-replicating cells must survive immunological and systemic clearance, while replicating cells must avoid uncontrolled replication, and both must traffic and be retained at treatment sites with high specificity over off-target tissues. These traits make pharmacokinetics and pharmacodynamics difficult to define. Thus, even if cells are engineered for programmable spatiotemporal control under in vitro settings, significant additional engineering will be required to ensure in vivo efficacy in multi-cellular and multi-tissue settings.

Third, living drug delivery vehicles must overcome risks associated with immunological toxicity. Systemic immune-mediated toxicities such as cytokine release syndrome and immune effector cell-associated neurotoxicity syndrome are two of the most common adverse events associated with engineered immune cells in human clinical trials [16,79,253]. These are caused by the uncontrolled and self-amplifying release of inflammatory cytokines by the patient’s native immune system and/or administered cells. Relatedly, allogenic human drug delivery vehicles risk triggering graft-versus-host disease. In addition, although bacterial drug delivery vehicles such as *M. pneumoniae* can be engineered for minimal immunogenicity [128], risks for sepsis remain if the required inoculum for therapeutic effect is significantly large and/or if such bacteria are replicative.

In principle, each of these biological hurdles can be solved by AI methods mature during the preclinical development phase. However, they illustrate some of the major challenges in translating in vitro to in vivo success.

### 6.2. Regulatory hurdles

Once a living drug delivery vehicle demonstrates preclinical success, it faces several new challenges before it can receive regulatory approval by health authorities such as the U.S. Food and Drug Administration and European Medicines Agency. First, candidate cell therapies must succeed in clinical trials that demonstrate safety and efficacy in humans. This is nontrivial because the financial costs and the ethics of evaluating drug candidates in human subjects limits the breadth of conditions in which drug candidates can be tested. Thus, clinical trial design is a critical bottleneck that can make or break not only a drug candidate but an entire category of drug classes. Several variables are important in designing clinical trials, including patient selection, clinical endpoints, biomarkers and biometrics, and dosing regimen. Patient-to-patient variability is a significant cause of clinical trial failures, which have spurred clinical trial design towards personalized precision medicine [254]. AI methods are becoming increasingly leveraged in clinical trial design [255,256], including adaptive clinical trials [257,258]. Conventional clinical trials may determine design parameters based on data from preclinical animal models of disease. However, because living drug delivery vehicles respond to biochemical cues in their environment, translating the performance of living drug delivery vehicles from animal models to humans is extremely difficult. To address some of these challenges, researchers are deploying AI approaches to bridge findings in animals to human [259-261]. In fact, at the time of this writing, the National Institutes of Health is currently in the process of shifting investments in animal model research to AI-based approaches [262,263].

### 6.3. Commercial hurdles

Even after a living drug delivery vehicle gains regulatory approval, it must be commercially viable for patients to benefit. A major component of commercial viability is whether a drug product can be cost-effectively produced and delivered to a patient. Some of the issues likely to become major challenges for living drug delivery vehicles include cost, scalable manufacturing, and storage stability. Because living drug delivery vehicles can only be expanded via cellular culture, they face unique expenses in following current good manufacturing practices (cGMP). For example, it may be cost prohibitive to create or acquire sterile culture media containing serum or recombinant proteins in large quantities. In addition, introduction of large gene circuits into living drug delivery vehicles by transformation or transduction may be difficult using current technologies. Moreover, robust large-scale biomanufacturing with minimal batch-to-batch variability may be difficult to execute, especially considering the selective pressures against engineered gene circuits [252,264]. Efforts are already underway to introduce AI methods into bioprocess engineering, but this is still an early area of development [265-268]. In addition, the delivery of living drug delivery vehicles from production facilities to patient end-users will be another critical challenge. While cold chain transport may be reasonable for bacterial drug delivery vehicles, cold chain logistics for human cell drug delivery vehicles also remain unclear.

## 7. Outlook and opportunities

Despite these hurdles, the future is bright for living drug delivery system engineering. For the first time, new AI and ML algorithms are coming online to keep pace with the rapid generation of large datasets by high throughput experimental platforms. In parallel, new synthetic biology technologies that can enable robust control over the composition and activities of biological molecules are advancing. Large-scale and systematic experimental studies are being performed in ways that can supply appropriate data for training advanced AI models that learn the design principles for engineering new biological parts and compositions with user-defined programmable behaviors. These collective advances are enhancing the ways in which we understand biology, thus enabling us to create biology [38].

In the coming years, AI and synthetic biology will synergistically accelerate the development of living drug delivery vehicles with advanced capabilities beyond delivering therapeutic cargo. Examples of these are already taking place in the world of engineered immune cell therapies. However, we envision that the scope of these engineered systems will go beyond current applications in clearing tumors or removing debris from damaged tissues. For example, many other human cell types are motile, including fibroblasts, pericytes, and endothelial cells which play important roles in angiogenesis and cardiovascular physiology. We hold that engineered versions of such cells can be functionalized to advance tissue regeneration by secreting regenerative factors (e.g., growth factors) or transdifferentiating into regenerative cell types [206].

To realize these opportunities, we propose three areas of future research to close gaps that currently rate-limit living drug delivery system engineering. First, there is a need for improved laboratory automation to establish fully end-to-end “self-driving laboratories” to further accelerate AI-driven DBTL of engineered cells [269-274]. Laboratory automation involves the use of robotics to massively scale simple experimental tasks in very high throughput. Currently, most laboratories that take advantage of laboratory automation still utilize human participation to analyze and interpret experimental data, generate hypotheses, and design subsequent experiments. We propose that as AI algorithms advance, these tasks can also be automated. Although most common examples of self-driving laboratories are from chemistry and material science research [275,276], principles of self-driving laboratories are already being adopted by synthetic biologists to design experiments that help characterize organisms [277], biological parts [278], and gene circuit compositions [279]. As large language model AI tools for performing hypothesis-driven research are becoming accessible [280], we envision that the adoption of self-driving laboratory practices will unlock biological insights beyond initial synthetic biology study designs and design objectives.

Second, there is a need for improved cell-type specific understanding of the circuit design principles specific to different candidate drug delivery vehicles. Nearly all synthetic biology activities currently take place in a small number of model organisms and cell types such as *E. coli* and HEK293 cells. Although these model systems have provided generalizable insights into the biology of living systems and standardized tools for synthetic biology, cell-type specific differences in cell physiology limit the predictability of how gene circuits designed and validated in one cell type will behave in a different cell type. For example, one of the greatest hurdles in synthetic biology in innate immune cells such as macrophages is the presence of elaborate DNA sensing machineries that inhibit the introduction of foreign DNA [281]. In addition, differential expression of gene regulatory elements such as native transcription factors, enhancers, repressors, and epigenetic modifiers will impact transgene expression. Moreover, differences in host cell resources such as ATP, amino acids, and other metabolic co-factors can limit gene circuit performance and/or induce biological stress responses that are not observed in model cell types [240,241,282-286]. Closing this gap may require adoption of self-driving laboratory practices to experimentally constrain the high-dimensional differences between cell types. However, AI approaches such as transfer learning can also help close this gap [52,287]. Transfer learning allows generalizable application of models trained in one environment into another environment through a shared knowledge source domain. Such approaches are widely adopted in fields such as single-cell RNA sequencing where experimental data can be sparse.

Finally, there is a need to better understand the fundamental design principles underlying how biological signals are processed to discover new ways of regulating the behaviors of engineered drug delivery vehicles. For example, although signaling dynamics are key regulators of cell fate in eukaryotic cells [288], very few synthetic biology studies specifically target cell signaling cascades as mechanisms for regulating gene circuit behavior [212]. This is in part due to the very large scale and complexity in signaling network topologies. First principles-based mathematical models are useful tools for predicting the behaviors of large-scale biological networks [289-292]. However, the significant knowledge gaps in signaling species, their connectivity, and their biochemical properties preclude the ability to comprehensively model cell signaling regulation of cell behaviors. A number of whole-cell modeling frameworks have recently been introduced for bacteria [293-297], however these approaches do not translate well for mammalian cells. Despite these limitations, new AI approaches are being developed to create models that can predict cellular responses to external cues without complete mechanistic understanding [298-301]. We envision that such models will inform the design of next-generation gene circuits that target signal transduction networks as a mode of regulating living drug delivery vehicle behaviors orthogonal to gene regulation alone.

## Figures and Tables

**Table 1:** Examples of living mammalian drug delivery vehicles.

Mammalian Cell-type	Cargo	Disease	References
Erythrocyte	Dexamethasone	Inflammatory bowel disease	68
	Enalaprilat	Cardiovascular disease	69
	PNA_PR2_	HIV infection	70
	IL-2	Cancer	71
	IL-12	Cancer	72
	L-asparaginase	Leukaemia	73
	Reteplase	Pulmonary edema	74
Macrophage	Indinavir	HIV infection	89
	Tirapazamine	Cancer	90
	Doxorubicin	Cancer	92
Th1	IFN-γ	Cancer	82
Th2	IL-4	Cancer	82
Treg	CD69	Cancer	82
γδT	IFN-γ	Cancer	82

**Table 2:** Examples of living bacterial drug delivery vehicles.

Bacterial Species	Cargo	Disease	References
*Bifidobacterium longum*	Oxyntomodulin	Obesity	[116]
	Manganese superoxide dismutase	Inflammatory bowel disease	[116]
	Trastuzumab scFv	Cancer	[117]
	Phenylalanine ammonia lyase, L-amino acid deaminase	Phenylketonuria	[118]
*Escherichia coli*	Microcin J25	*S. typhimurium* infection	[119]
	Glucagon-like peptide 1	Obesity	[120]
	CD47 nanobody	Cancer	[121]
	PD-L1, CTLA4, NPI, Stx2 nanobodies	Inflammatory bowel disease	[122]
	IL-10	Inflammatory bowel disease	[123]
*Lactococcus lactis*	β-lactamase	*Clostridioides difficile* infection	[124]
	Anti-TNFα scFv	Inflammatory bowel disease	[125]
	VEGF	Diabetic wound	[126]
	CSF-1, FGF-2, IL-4	Diabetic wound	[127]
	IL-10	*P. aeruginosa* infection	[128]
*Mycoplasma pneumoniae*	Dispersin B, lysostaphin	*S. aereus* infection	[129]
	IL-10	*P. aeruginosa* infection	[130]
	PelAh, PslGh, AI-II’, dispersin B	*S. aureus, P. aeruginosa* co-infection	[131]
*Pseudomonas aeruginosa*	MyoD	Myogenesis	[132]
	Ovalbumin antigen	Cancer	[133]
	TRP-2, gp100, MUC18	Cancer	[134]
	HlyE	Cancer	[135]
	Hepatitis B surface antigen, hepatitis C virus	Hepatitis	[136]
*Salmonella typhimurium*	Cp53	Cancer	[137]
	Senecavirus A	Cancer	[138]
	CCL21	Cancer	[139]
	GM-CSF, IL-7	Cancer	[140]
	IL-18	Cancer	[141]
	IL-2	Cancer	[142]
	HlyE, CCL-21, Bit1 cell death domain	Cancer	[143]

## Data Availability

No data was used for the research described in the article.

## HIGHLIGHTS

- Human and bacterial cells can be engineered into living drug delivery vehicles.
- AI can be leveraged to design synthetic receptors against novel targets.
- AI can be leveraged to design gene regulatory elements with predictable behaviors.
- AI can be leveraged to design programmable gene circuit compositions.
